# Chromium(III) Removal from Nickel(II)-Containing Waste Solutions as a Pretreatment Step in a Hydrometallurgical Process

**DOI:** 10.3390/ma15186217

**Published:** 2022-09-07

**Authors:** Milena Kostrzewa, Katarzyna Staszak, Dobrochna Ginter-Kramarczyk, Izabela Kruszelnicka, Wojciech Góra, Marek Baraniak, Grzegorz Lota, Magdalena Regel-Rosocka

**Affiliations:** 1 Institute of Chemical Technology and Engineering, Faculty of Chemical Technology, Poznan University of Technology, ul. Berdychowo 4, 60-965 Poznan, Poland; 2 Department of Water Supply and Bioeconomy, Faculty of Environmental Engineering and Energy, Poznan University of Technology, ul. Berdychowo 4, 60-965 Poznan, Poland; 3 Institute of Chemistry and Technical Electrochemistry, Faculty of Chemical Technology, Poznan University of Technology, ul. Berdychowo 4, 60-965 Poznan, Poland

**Keywords:** chromium removal, nickel, waste solutions, hydrometallurgy, precipitation, adsorption, ion-exchange resin

## Abstract

This paper presents Cr(III) removal from nickel sulfate waste solutions as a pretreatment step for the modification of hydrogen storage alloys. Adsorption with two cation exchange resins, Dowex G26 (strongly acidic) and MAC-3 (weakly acidic), and precipitation with various solutions were chosen as simple operations for Cr(III) removal from waste solutions. The adsorption of Cr(III) was investigated for both model and real waste nickel solutions. Dowex G26 appeared to be more efficient in Cr(III) removal (R_Cr(III)_ from 43 to 80%) than MAC-3 (R_Cr(III)_ from 40 to 53%). However, the adsorption from multi-component solutions (presence of Co(II), Ni(II) and Cr(III)) showed no selectivity in Cr(III) adsorption in comparison to those of Co(II) and Ni(II). Cr(III), Ni(II) and Co(II) were removed at a comparable level (30–36%) from a three-component solution of 10 g/dm^3^ of each metal ion, and a 56–72% removal of these ions was achieved from the real solution. Therefore, the precipitation of Cr(III) was carried out from a real waste nickel solution to compare its performance with adsorption. The best precipitation solution appeared to be 3 and 30% NaOH due to the quantitative precipitation of Cr(OH)_3_ at pH 5 and relatively small co-precipitation of Ni(II) and Co(II) hydroxides (P_Co(II)_ = 20–52%, P_Ni(II)_ = 0–54%). Based on the results of the research, it can be concluded that precipitation with a NaOH solution is an efficient pretreatment operation of an electrolyte for further steps of the hydrometallurgical process of nickel electrodeposition and appears to be more selective in the elimination of Cr(III) than adsorption with Dowex G26 resin.

## 1. Introduction

Increased ecological awareness and market needs have made the recovery of metals from waste solutions an important research and practical issue in recent years, especially in view of the decreasing availability and content of metals in natural deposits. In addition, as a result of the growing number of various types of waste products, the methods of their management and use as sources of valuable raw materials, e.g., metals, are sought not only to replace natural deposits of raw materials but also to contribute to the reduction in the amount of collected waste [1]. 

Stainless steels and alloys are among the most important types of materials used in the modern world. For example, global stainless steel production in 2020 exceeded 50 million metric tons [2]. This, in turn, is related to the availability of waste materials of these types for the recovery and recycling of alloys and their individual components. These types of steel, in addition to iron, contain chromium and many other elements, including valuable non-ferrous metals. One of them is nickel. Nickel is widely used in metallic forms, alloys and compounds [3,4]. One of its most important areas of application is currently in chemical power sources. The market for chemical power sources is continuously growing and is one of the basic elements of the development of civilization [5].

The dominant batteries are lead-acid (Pb-A) and lithium-ion (Li-ion) systems; however, although Ni-MH batteries are inferior to Li-ion systems in terms of capacity, they are safer than them because of the water electrolyte they contain. In this type of cell, hydrogen is stored in a reversible way. The materials used as negative electrodes in Ni-MH systems are multi-component stoichiometric and non-stoichiometric alloys of the following types: (i) AB_5_ Hauck phase, (ii) AB_2_ Laves phases, (iii) AB type CsCl-CrB, (iv) A_2_B type Ti_2_Ni, (v) amorphous alloys [6]. Nickel is an important component of the vast majority of these alloys, which leads to a major economic significance of this metal in electrochemical applications. These alloys, in addition to their ability to reversibly absorb hydrogen, have catalytic properties that can be used in fuel cells with direct oxidation of borohydrides (direct borohydride fuel cell, DBFC) [7]. These types of systems have high electric parameters and are cheaper than other fuel cells because of the possibility of replacing platinum as a catalyst with other metals, for example, nickel. Experiments indicate that the deposition of some amount of nickel on these alloys causes an increase in the efficiency of the systems [7,8,9]. Ni(II)-containing waste solutions obtained as a result of the leaching of stainless steel appear to be a valuable source of this metal for the modification of the alloys; however, they are contaminated with a significant amount of Cr(III) [10], which does not exhibit such high catalytic properties for these reactions.

It should be noted that the use of electrochemical methods for metal deposition requires a recognition of many factors, especially from waste solutions that usually contain many electroactive components. One of them, among others, is the presence of different metal ions that can be co-deposited. Selective metal deposition, in this case, depends not only on the difference in standard potential values between the metals but also on the relative ratio of their concentrations, the presence of complexing substances and substances that show adsorption abilities on the surface. Therefore, nickel deposition from solutions after the leaching of alloy steels should be performed from solutions containing relatively high concentrations of nickel and low concentrations of chromium. Only the use of such electrolytes should allow the selective recovery of nickel and its use in the modification of electrode materials [11,12].

A proposal for the modification of hydrogen storage alloys with electrodeposited nickel obtained from Ni(II)-containing waste solutions fits perfectly into a new model of the economy—the circular economy. Thanks to such an approach, it is possible to close the product life cycle and switch from the linear economy model (raw material acquisition–production–use–waste disposal) to the circular model (production–use–reuse of waste as a raw material in the next production cycle). 

The methods of treatment of wastewater containing chromium have been known to meet increasingly strict consent levels and regulations. In the environment, chromium occurs mainly in trivalent and hexavalent forms. Hexavalent chromium Cr(VI) is toxic and carcinogenic, while the element in its trivalent state Cr(III) is an essential nutrient for plant and animal metabolism in trace amounts [13]. However, Cr(III) has also been shown to be a potential hazard, especially in the aquatic environment. Conventional chromium removal methods include adsorption, chemical precipitation, ion exchange, solvent extraction, biosorption, membrane separation and electrochemical methods [14,15,16,17,18]. Moreover, solid phase extraction has been proposed for trace metal removal or preconcentration in various analytical methods or from environmental samples [19,20,21].

The removal of the trivalent form of chromium has been reported mainly from tanning wastewater. Chemical precipitation (CP) is the most widely used method in Cr-contaminated wastewater treatment due to its maturity in technique, simplicity of equipment, operational flexibility and ability to recover Cr(III). The most common method is chemical precipitation as hydroxides. Otherwise, researchers propose to use activated carbons to effectively adsorb Cr(III) from tanning effluents of pH about 3 [22,23]. For more acidic solutions (pH ~1 and higher), cation exchange resins have been reported to efficiently adsorb Cr(III) [24,25]. However, in terms of the selectivity of cation exchange resins toward Cr(III) in wastewater from the aluminum anodic plating process (pH 3–4), it turned out that aluminum and nickel adsorption with Amberlite IR120 or Lewatit TP207 is preferred over chromium [26]. In addition, natural zeolite appeared to be an inefficient material for Cr(III) and Ni(II) adsorption. Other cation-exchange resins (Amberlite IRN77 and Purolite C160) were proposed for Cr(III) adsorption from model nitrate solutions at pH 4 [27]. However, the research involved only model solutions, and it would be difficult to transfer the results to real wastewater systems. In addition, a chelating resin Amberlite XAD-4 functionalized with salicylic acid by coupling through an azo spacer has been proposed for the preconcentration of various metal ions, including Cr(III) [28]. Moreover, it should be mentioned that nanoparticles, such as low-cost bare magnetic Fe_3_O_4_, have been proven to perform an ultra-high Cr(III) capture capacity under high selectivity from tanning wastewater at optimal pH 8, i.e., after transformation of Cr(III) ions into Cr(OH)_3_ colloid [29]. Other magnetic mesoporous microspheres Fe_3_O_4_@nSiO_2_@mSiO_2_ modified with triacetic acid NTA (FNMs-NTA) exhibited excellent Cr(III) adsorption capacity in the presence of inorganic cations and/or complexing agents in the model solutions, as well as remarkable separation and regeneration performance [30]. The commercial resin, Amberlite IRN77, was also modified by coating with magnetite nanoparticles Fe_3_O_4_ and reached a higher maximum adsorption capacity (32.7 mg/g) than Amberlite IRN77 itself (23.9 mg/g) [31]. The advantageous property of the magnetic sorbents is the ease of separation of the material after adsorption by an external magnetic field.

Thus, the literature reports confirm that chromium ions can be removed by adsorption, with an important factor being the correct choice of the sorbent as well as the process conditions. Therefore, it is important to investigate different groups of resins in this regard.

In our previous research, the precipitation of Cr(III) hydroxide with a concentrated NaOH solution was proposed to remove contaminating Cr(III) from the leach solution [10]. Subsequently, Co(II) was separated from Ni(II) by liquid–liquid extraction to produce two solutions of these metals that could potentially be used for electrowinning of cobalt and nickel. As Co(II) extraction is carried out at pH~5, Cr(III) must be removed to avoid both the formation of a Cr(OH)_3_ deposit during extraction and disturbing the effective extraction of Co(II). 

The research conducted focuses on the removal of Cr(III) from Ni-containing solutions. The novelty of the studies lies in the involvement of this pretreatment step in the preparation of real Ni(II)-containing solutions for the manufacturing of hydrogen storage materials by Ni electrodeposition. It should be emphasized that this research focuses on the conventional methods of Cr(III) removal (adsorption/ion exchange and precipitation), as the development of a hydrometallurgical process for nickel purification is ultimately planned. Thus, it is important to involve in the process established, well-known and simple techniques to make the process easily operated and economical. The adsorption of Cr(III) was investigated for both model and real leach solutions, while the precipitation of Cr(III) was performed from a real solution to compare its performance with adsorption and to choose the best operation for the preparation of an electrolyte for further steps of the hydrometallurgical process of nickel electrodeposition.

## 2. Materials and Methods

### 2.1. Apparatus and Chemicals

Real undiluted pregnant leach sulfate solutions (PLS), received after the treatment of stainless steel scraps, were supplied by one of the Polish companies involved in waste treatment. These real solutions were taken for investigation of Cr(III) removal. The content of the main compounds in the PLS before diffusion dialysis (DD) and after (for more details, see Section 3.1) is presented in Table 1.

The following reagents were used for analytical and adsorption purposes: NaOH (volumetric solution or microgranulate), HNO_3_ (65%), HCl (35–38%), acetone (p.a.) purchased from Avantor Performance Materials Poland S.A. (Gliwice, Poland), H_2_SO_4_ (95%) from Chempur (Piekary Slaskie, Poland). The chemicals used in this study for precipitation (Avantor Performance Materials Poland S.A.) were as follows: lime solution (10% CaO), sodium hydroxide (15% NaOH) and sodium hydrogen carbonate (10% NaHCO_3_). All reagents were of analytical reagent grade and were used as received without purification.

Two commercially available cation exchange resins (acidic) were chosen for adsorption experiments, Dowex G26 and Dowex MAC-3 (Sigma Aldrich, Poznan, Poland). The properties of the resins used are shown in Table 2.

### 2.2. Instrumentation

An atomic absorption spectrometer (AAS, ContrAA 300, Analytik Jena, Jena, Germany) was used for the measurement of metal ion concentrations in aqueous samples at the following wavelengths: 214, 232, 240, 248, 285, 324, 359 nm for Zn(II), Ni(II), Co(II), Fe(III), Mg(II), Cu(II) and Cr(III), respectively. Inductively coupled plasma optical emission spectrometry (ICP-OES) was applied to determine the concentration of Al(III) in the aqueous solutions at 396.2 nm. Appropriate dilutions were prepared to ensure that the concentrations of the metal ion solutions were in the range of the analytical curve. The concentration result is an average of three measurements. 

Concentrations of H^+^ and Cl^−^ ions were controlled by potentiometric titration with solutions of 0.1 M NaOH or 0.1 M AgNO_3_, respectively, using Metrohm 703 TiStand equipment (Herisau, Switzerland). The concentration of SO_4_^2−^ was measured by a capillary isotachophoresis electrophoretic analyzer EA 100 (Villa Labeco, Spisska Nova Ves, Slovakia).

### 2.3. Adsorption Procedure

Solutions of the one-component model with appropriate concentrations of Cr(III) were prepared: 2, 10, 40 g/dm^3^ and H^+^ content equal to 3 M. These compositions were chosen to obtain the greatest possible similarity to the composition of real leach solutions, the composition of which is given in Table 1. Three-component model solutions containing Cr(III), Ni(II), Co(II) (0.1, 0.5, 1, 2, 10 g/dm^3^) in 3 M H^+^ were prepared to investigate the adsorption selectivity.

For batch adsorption, 2 cm^3^ of the model or real solution was added to the 15 cm^3^ test tubes with a proper amount (30, 50, 100, 250, 500 and 2000 mg) of the resin G26 or MAC-3 (properties shown in Table 2). Samples prepared in this way were placed on a GFL rotary shaker and shaken for 18 h. During shaking, 0.2 cm^3^ samples were taken for analysis of Cr(III) content after 30, 60, 120, 150, 1080 or 1440 min of adsorption. Selected batch adsorption experiments were performed twice, and the error did not exceed 11%. 

Fixed-bed column adsorption was carried out in a glass column (18 mm of inner diameter, 200 mm long, 55 cm^3^ bed volume) filled with 36 g of resin on a dry-weight basis. The bed height was 170 mm. The inlet flow rate was controlled by a peristaltic pump (Lead Fluid, model BT103S, China) and was equal to 4.4 cm^3^/min. An amount of 200 cm^3^ of Cr(III)-containing solution was circulated through the column for 24 h. Samples of 0.1 cm^3^ volume were collected from the container at the bottom of the fixed-bed column at different time intervals, and the concentration of metal ions was determined by AAS. Adsorption was followed by desorption with 1.5 M H_2_SO_4_. Finally, the adsorption bed was washed with 100 cm^3^ deionized water to prepare it for further adsorption experiments. The conductivity and pH values during the experiment were continuously recorded by the multi-functional Elmetron device CX-601 (Zabrze, Poland). All the adsorption experiments were carried out at 23 ± 2 °C.

The following relationships were used to describe adsorption:

− sorption capacity, *q_e_* in mg/g:

(1) $$q_{e}=\frac{m_{Cr\_0}-m_{Cr\_e}}{m_{s}}$$

− sorption capacity under non-equilibrium conditions, *q_t_* in mg/g:

(2) $$q_{t}=\frac{m_{Cr\_0}-m_{Cr\_t}}{m_{s}}$$

− percentage removal of Cr(III) ions, *R*:

(3) $$R=\frac{m_{Cr\_0}-m_{Cr\_t}}{m_{Cr\_0}}\cdot 100\%$$

where $m_{Cr\_0}$—the initial mass of Cr(III) in the aqueous phase in mg, *m_Cr_e_*—the equilibrium mass of Cr(III) in the aqueous phase in mg, *m_Cr_t_*—the mass of Cr(III) in the aqueous phase after *t* in mg and *m_s_*—the mass of dry sorbent.

Moreover, the four isotherms models were used for the calculation:

− Langmuir isotherm:

(4) $$q_{e}=\frac{k_{L}Q_{0}C_{e}}{1+C_{e}k_{L}}$$

where *Q*_0_ is the monolayer adsorption capacity (mg/g), *k_L_*—the Langmuir constant (related to the free energy of adsorption) (dm^3^/mg), *C_e_*—equilibrium concentration of Cr(III) ions in the aqueous phase.

− Freundlich isotherm:

(5) $$q_{e}=k_{F}C_{e}^{1/n}$$

where *k_F_* (mg^1−1/n^ dm^3/n^/g^1^) and *n* are the constants of the Freundlich isotherm related to the adsorption capacity of the adsorbent and the surface heterogeneity, respectively.

− Temkin isotherm:

(6) $$q_{e}=\frac{RT}{b_{T}}lnAC_{e}$$

where *A* (dm^3^/g) and *b_T_* (J/mol)—the Temkin constants, *R*—the universal gas constant (8.314 J/mol K), *T*—the temperature (K).

− The Dubinin–Radushkevich isotherm:

(7) $$q_{e}=q_{m}e^{k_{DR}\varepsilon^{2}}$$

where *ε*, the adsorption potential (J/mol), is given by

(8) $$\varepsilon =RTln\left[1+\frac{1}{C_{e}}\right]$$

*k_DR_* (mol^2^ J^2^) is the parameter associated with adsorption energy, and *q_m_* (mg/g) is the maximum adsorption capacity. From the Dubinin–Radushkevich isotherm, it is also possible to calculate the mean free energy for departure of molecules from their adsorption site to the infinity, *E* (J/mol):

(9) $$E=\frac{1}{\sqrt{2k_{DR}}}$$

Pseudo-first- and second-order equations are used to describe adsorption kinetics (PFO and PSO, respectively) according to the equations:

− PFO:

(10) $$r_{I}=\frac{dq_{t}}{dt}=k_{1}\left(q_{e}-q_{t}\right)$$

− PSO:

(11) $$r_{II}=\frac{dq_{t}}{dt}=k_{2}{\left(q_{e}-q_{t}\right)}^{2}$$

The procedure for the fitting of the kinetic parameters was based on the application of a non-linear least squares minimization algorithm using a Matlab program to estimate the derivative of the double exponential function $dq_{t}=a\cdot exp\left(b\cdot t+c\right)+d$:

(12) $$\frac{dq_{t}}{dt}=a\cdot b\cdot exp\left(b\cdot t+c\right)$$

### 2.4. Precipitation Procedure 

Precipitation studies were carried out in batch mode. The pH was adjusted to the desired values within the range of 3 to 5 by dropwise addition of one of the alkaline solutions. Alkaline solutions of NaOH (3 or 30%), CaO (30%) and NaHCO_3_ (30%) were examined. A precipitation solution was added in small portions to a 50 cm^3^ aliquot of sulfate PLS to obtain the desired pH (3, 4 or 5). The pH during precipitation was controlled by Elmetron CP-411 (Poland). When the system reached equilibrium, it was left under the stirring conditions for an hour (to age the precipitate produced). After this time, the samples were centrifuged, and the resulting liquid was analyzed. Selected precipitation experiments were performed twice, and the error did not exceed 5%. All the precipitation experiments were carried out at 23 ± 2 °C.

The yield of precipitation of metal ions (*Me*), *P,* was calculated according to the following equation:

(13) $$P=\frac{m_{Me\_0}-m_{Me\_a}}{m_{Me\_0}}\cdot 100\%$$

where $m_{Me\_0}$—the initial mass of metal ions in the aqueous phase, *m_Me_a_*—the mass of metal ions in the aqueous phase after precipitation.

## 3. Results and Discussion

### 3.1. General Information about the Proposed Process

**Figure 1:**
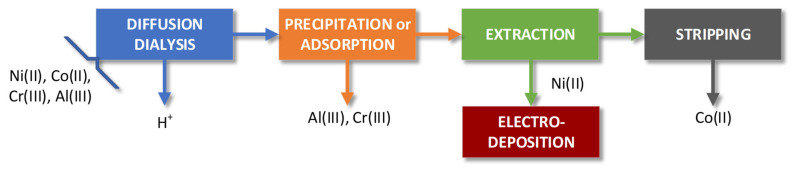
Scheme of the proposed hydrometallurgical process.

To selectively separate Ni(II) from waste solutions containing other ions, such as Cr(III), Al(III), Co(II), Fe(II), Fe(III) and/or (III), chlorides, sulfates, etc., a hydrometallurgical process was proposed (Figure 1). It included operations such as diffusion dialysis (DD), Cr(III) removal by precipitation (P) or adsorption (A), extraction of Co(II) into the organic phase (E), Co(II) stripping from the loaded organic phase (S) and the organic phase regeneration and reuse. Ni(II)-containing raffinate after extraction was required for the final stage of the nickel recovery, i.e., for nickel electrodeposition on the cathodes. 

Diffusion dialysis was carried out in the typical way described in the previous publication [10]. This work focuses on the removal of Cr(III) from the feed after DD to prepare an aqueous solution for Ni(II)/Co(II) separation with liquid–liquid extraction. If Cr(III) is present in the aqueous phase during Co(II) extraction, which is performed at pH 5–5.2, a stiff precipitation of Cr(III) hydroxide forms, which prevents the extraction and phase separation. To avoid problems during extraction, Cr(III) must be removed prior to the next steps of the process. 

Adsorption (ion exchange) and precipitation seem to be the easiest operations to achieve the goal of efficient Cr(III) removal. 

### 3.2. Cr(III) Removal by Adsorption 

#### 3.2.1. Batch Adsorption from One-Component Model Solutions

As indicated in the scientific literature, Cr(III) in acidic sulfate solutions can form cationic dimers [(H_2_O)_3_Cr(OH)(SO_4_)(OH)Cr(H_2_O)_3_]^2+^; however, [Cr(H_2_O)_6_]^3+^ can also occur [22]. The cationic species could be retained in acidic resins as a result of cation exchange:

(14) $$2RCOOH+{\left[Cr_{2}{\left(OH\right)}_{2}SO_{4}\right]}^{2+}↔{\left(RCOO\right)}_{2}\left[Cr_{2}{\left(OH\right)}_{2}SO_{4}\right]+2H^{+}$$

(15) $$3RCOOH+Cr^{3+}↔{\left(RCOO\right)}_{3}\mathrm{Cr}+3H^{+}$$

**Figure 2:**
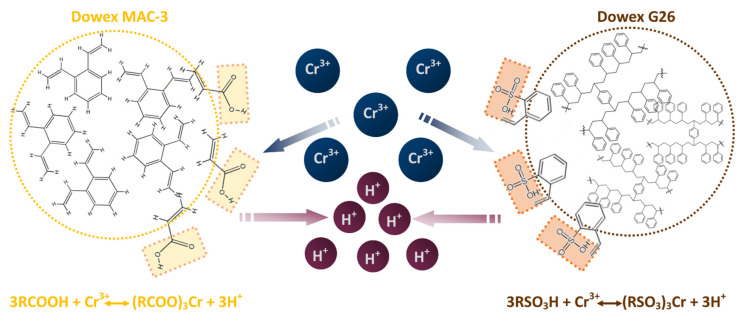
The graphical representation of the mechanism of Cr(III) adsorption with Dowex G26 and MAC-3.

Therefore, acidic resins were chosen for the adsorption of Cr(III) from acidic leach solutions, and the graphical representation of the Cr(III) adsorption mechanism with Dowex G26 and MAC-3 is shown in Figure 2.

Moreover, from Equations (14) and (15), it can be concluded that an acid will effectively desorb the metal ions in reaction reverse to adsorption. Not only will Cr(III) be recovered from the resin, but the adsorbent will also be regenerated. 

Batch adsorption was studied to check if the selected resins were efficient in Cr(III) adsorption from acidic solutions. At first, the sorption from one-component model solutions was performed, and then, three-component model solutions and, finally, a real waste solution were also contacted with the more efficient resin, chosen on the basis of the results for model solutions. 

**Figure 3:**
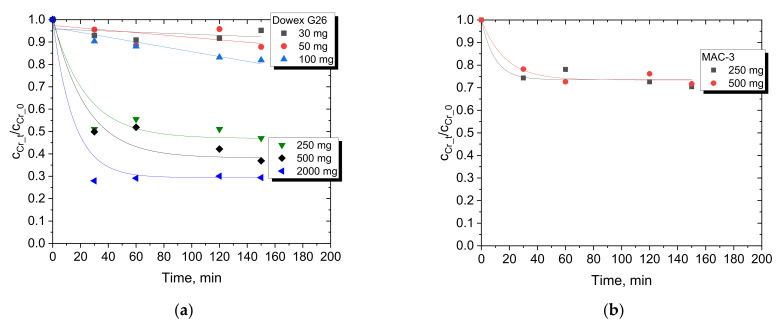
Cr(III) adsorption from model sulfate solution with various amounts of (**a**) Dowex G26 and (**b**) MAC-3 resins (feed: 2 g/dm^3^ Cr(III), 3 M H^+^, L/S = 1/250 cm^3^/mg).

**Figure 4:**
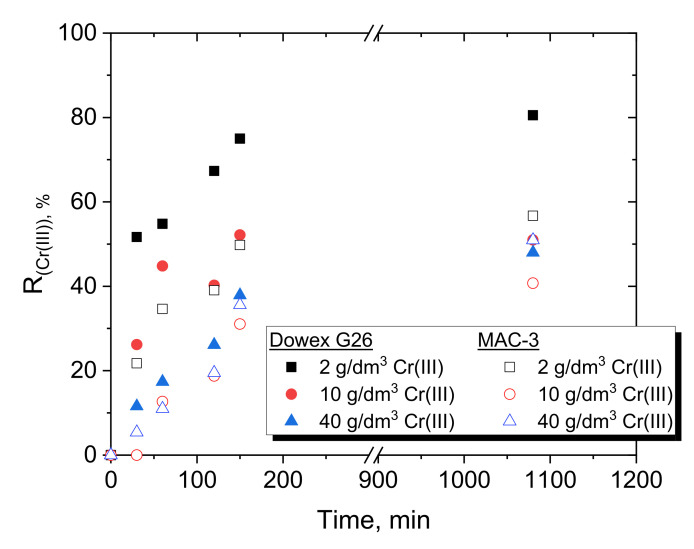
Comparison of Cr(III) removal over time with 500 mg of Dowex G26 (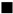, 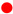, 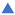) or MAC-3 (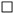, 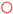, 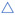) from feeds of various Cr(III) concentrations (3 M H^+^, L/S = 1/250 cm^3^/mg).

The change in Cr(III) concentration and Cr(III) removal in the course of adsorption is shown in Figure 3 and Figure 4, respectively.

As expected, with increasing amounts of Dowex G26 resin (decreasing L/S ratio from 1/15 to 1/1000 cm^3^/mg), the concentration of Cr(III) in the feed is reduced even by two-thirds for 2000 mg of the resin used. However, there is no large difference in decreasing Cr(III) concentration in the feed between the use of 500 and 2000 mg of Dowex G26, and therefore, 500 mg was chosen for most adsorption experiments. Based on the preliminary results for Dowex G26 (Figure 3a), only two amounts of resin were investigated for MAC-3, i.e., 250 and 500 mg (Figure 3b). It is visible in both Figure 3 and Figure 4 that Cr(III) adsorption is more efficient with Dowex G26 than with MAC-3. Especially when the initial concentration of Cr(III) is low, Dowex G26 (L/S = 1/250 cm^3^/mg) retains 80% of the metal ions, while MAC-3 adsorbs more than 50%. When 40 g/dm^3^ Cr(III) was present in the feed, Dowex G26 and MAC-3 were able to remove comparable amounts from the aqueous solution, close to 50%. It should also be noted that 150 min is enough to achieve equilibrium (the Cr(III) concentration does not change) for adsorption with both resins in the amount range of 250–2000 mg.

**Figure 5:**
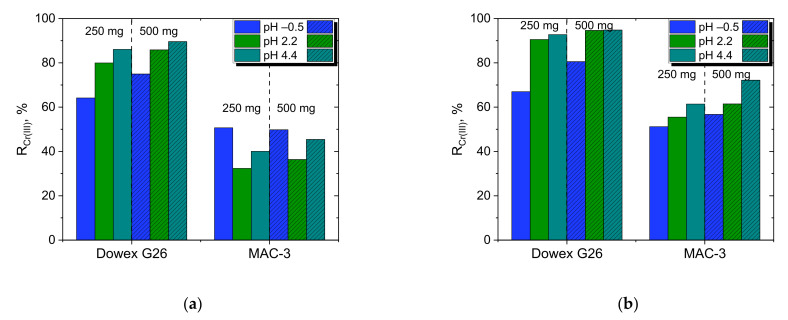
Effect of pH on Cr(III) adsorption from model solution with 250 or 500 mg of Dowex G26 or MAC-3 after (**a**) 150, (**b**) 1080 min (18 h) of batch adsorption (feed: 2 g/dm^3^ Cr(III), various pH, L/S = 1/125 or 1/250 cm^3^/mg).

Because from a practical point of view, the influence of pH on adsorption efficiency is important, the effect of pH on the adsorption of Cr(III) by Dowex G26 and MAC-3 was studied using a model solution containing 2 g/dm^3^ Cr(III) at pH −0.5 (3 M H^+^), 2.3, 4.4, and the results are shown in Figure 5. 

Typically, for cationic exchange resins, the capture of metals increases with increasing pH. It is worth mentioning that even at high H^+^, i.e., low pH, the adsorption efficiency of Cr(III) with Dowex G26 exceeds 60% after 2.5 h of contact. Following the observations of Alguacil for the acidic resin Amberlite 200 [24], it can be assumed that Dowex G26 also has the potential to be applied to remove Cr(III) even from solutions of high acidity. Unlike Dowex G26, weak acidic MAC-3 does not show good Cr(III) removal properties after 150 min of contact (maximum 45% Cr(III) removal). After 1080 min of phase contact, the adsorption efficiency with MAC-3 increases to even 65% at pH 4.4 and L/S = 1/250 cm^3^/mg (500 mg of resin). This again confirms that this resin is not suitable for the tested system.

#### 3.2.2. Kinetic and Isothermal Parameters of a One-Component Adsorption System

**Figure 6:**
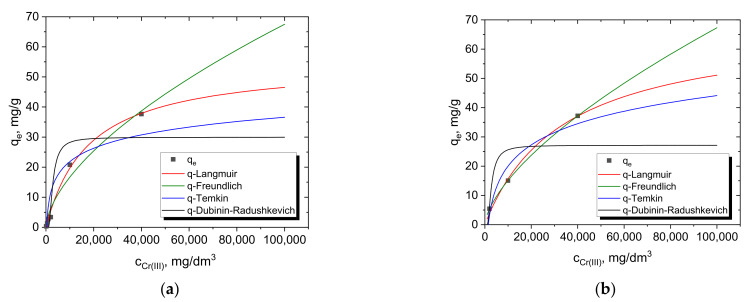
Fitting of Langmuir, Freundlich, Temkin and Dubinin–Radushkevich isotherms with the experimental data for the system with 500 mg of sorbents (**a**) Dowex G26; (**b**) MAC-3.

The equilibrium and kinetic studies of Cr(III) adsorption from one-component model solutions allowed the performance and efficiency of the operation to be determined. According to the theoretical assumptions, the efficiency of the adsorbent was determined from equilibrium studies based on the Langmuir, Freundlich, Temkin and Dubinin–Radushkevich isotherms [32]. The calculated parameters are summarized in Table 3, while the comparison of all isotherms with the experimental data is presented in Figure 6. On the other hand, the kinetic studies allowed the estimation of the removal rate of chromium ions from the system by applying the pseudo-first-order and second-order equations (PFO, PSO) (Table 4 and Table 5).

Based on the data presented in Table 3, it can be concluded that of the four isotherm models, the highest *R*^2^ values were obtained for the Langmuir isotherm (0.993–0.995) and slightly lower for the Freundlich isotherm (0.973–0.997). From the data, it can also be concluded that favorable sorption occurs with a value of 1/n less than 1 [33]. A similar relationship was observed for the vanadium [34] and copper [35] sorption process on the Dowex G26 resin. The Temkin and Dubinin–Radushkevich models cannot be applied for the description of these adsorption systems due to the fact that the equilibrium data obtained did not show a good fit with the model (R^2^ in the range of 0.8 to 0.6 for MAC-3 for the D-R isotherm). It should also be noted that the estimated energy values of the *D*-*R* Equation (7) indicate the physical nature of adsorption, according to the physical rule (*E* < 8 kJ/mol) or chemical nature (E = 8–16 kJ/mol) [36], but the low values of *R^2^* do not allow this to be confirmed unequivocally. 

The results of Cr(III) removal with Dowex G26 and MAC-3 obtained in the present studies are compared in Table 6 with the adsorption values reported for acidic resins by other researchers. Most of the investigation deals with model solutions of low Cr(III) content (less than 0.5 g/dm^3^) and mild acidity; therefore, it is difficult to draw unambiguous conclusions. However, it should be noted that Dowex G26 reveals a very high adsorption capacity, the highest among all commercial cation exchange resins. Only a synthesized polyampholyte PVC-(SO_3_H)-(NH) resin was reported to adsorb 206 mg Cr(III) per 1 g of the resin [37], which is the highest value of the resins presented in Table 6.

**Figure 7:**
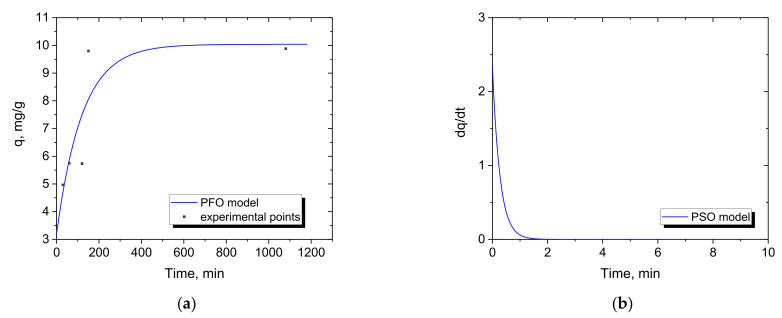
Experimental data for Cr(III) adsorption with 250 mg of MAC-3 with double exponential function for calculation of (**a**) PFO and (**b**) PSO kinetic models and the adsorption rate (feed: 2 g/dm^3^ Cr(III) solution at pH −0.5).

Based on the experimental data on the changes in concentration of Cr(III) in the system during the adsorption, the kinetic dependence, shown for an exemplary system in Figure 7, was determined. According to Equation (12), a double exponential function describing this dependence was determined, which, after differentiation, allowed the estimation of the kinetic parameters of the systems in the pseudo first and second order (Table 4 and Table 5). The parameters *a*–*d* of the fitted Equation (12) are presented in Table A1 and Table A2 in Appendix A.

The results show differences in the adsorption kinetics for the two sorbents tested. In the case of Dowex G26, significantly higher *R*^2^ values were obtained for the PFO model than for MAC-3 for the PSO model, regardless of the initial concentration of Cr(III), the amount of sorbent and the pH. Thus, it could be concluded that chemical sorption is the rate-limiting step involving valence forces through the sharing or exchange of electrons between MAC-3 and Cr(III). In contrast, for Dowex G26, the PFO model indicates a physisorption mechanism, where the adsorption rate depends on the sites available in the adsorbent [40].

#### 3.2.3. Batch Adsorption of Cr(III) from Multi-Component Solutions

Three-component model solutions of various concentrations of Cr(III), Ni(II) and Co(II) were contacted with 500 mg of Dowex G26 and MAC-3. The results of Cr(III) removal from the feed solution (3 M H^+^) are presented in Table 7. 

Although the adsorption from the three-component solution appeared not to be selective toward the removal of Cr(III) from the solution, finally, the real waste solution was also contacted with Dowex G26, chosen based on the results for the model solutions being more efficient than the MAC-3 resin. The removal of Cr(III) was not selective, and the ions of the three metals were removed at a comparable level (30–36%) from the three-component solution of 10 g/dm^3^. The percentage removal from the real solution reached higher values than from the three-component model solutions because 2000 mg of resin was used compared to 500 mg in the case of the model solutions. Therefore, the Cr(III), Ni(II) and Co(II) removal values (56–72%) were twice those of the model solutions. The exception was visible during the removal of Ni(II) from the 2 g/dm^3^ metal ion solution. The removal of Ni(II) from the beginning was the highest and finally reached more than 80%. Nevertheless, the adsorption results obtained for the multi-component solutions show that the use of cation exchange resin, such as Dowex G26, as a pretreatment step for the modification of hydrogen storage alloys would be problematic because of the loss of large amounts of valuable Ni(II) and Co(II) from the aqueous solutions. Similar reduction in Cr(III) adsorption was observed in the presence of Al(III) and Ni(II) at pH 3.5 when Amberlite IR120 and Levatit TP207 were used as cation exchange resins (Table 4) [26]. After 48 h of contact, 97% Al(III) was adsorbed, while 66% Ni(II) and 18% Cr(III) were retained on strongly acidic Amberlite IR120, and only 17% Ni(II) and 12% Cr(III) on weakly acidic Levatit TP207. 

#### 3.2.4. Column Adsorption 

Cr(III) adsorption was investigated in a fixed-bed column for Dowex G26 resin. The adsorption from three solutions was compared, i.e., single- and three-component model solutions and the real PLS after DD. The initial concentrations of metal ions in the model solutions (1 g/dm^3^ of ions of each metal in 1.5 M H_2_SO_4_) were lower than in the real PLS after DD (24.55 g/dm^3^ Cr(III), 37.17 g/dm^3^ Ni(II), 21.37 g/dm^3^ Co(II) in 1.73 M H_2_SO_4_).

**Figure 8:**
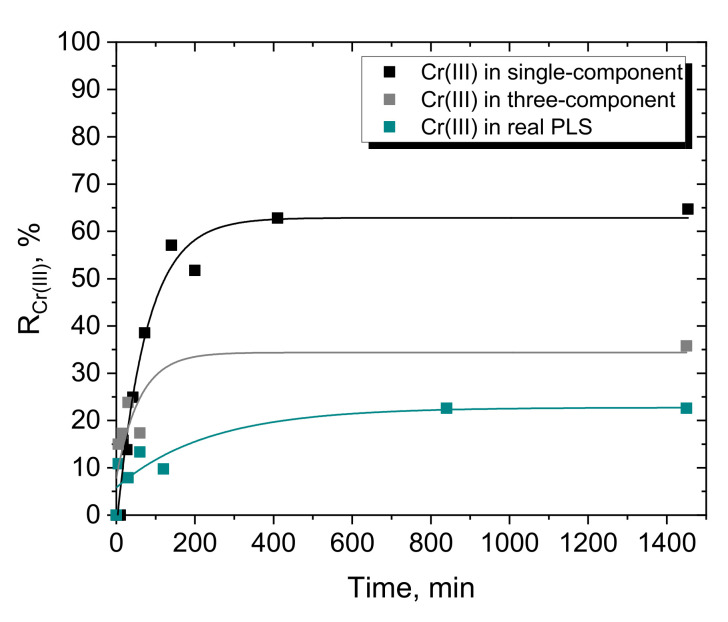
Removal of Cr(III) in the fixed-bed column with Dowex G26 resin from (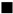) single-component model solution (1 g/dm^3^ Cr(III), 3 M H^+^), (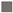) three-component model solution (1 g/dm^3^ Cr(III), 1 g/dm^3^ Ni(II), 1 g/dm^3^ Co(II), 3 M H^+^), (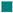) real PLS after DD (24.5 g/dm^3^ Cr(III), 37.2 g/dm^3^ Ni(II), 21.4 g/dm^3^ Co(II), 3.46 M H^+^) (flow rate 4.4 cm^3^/min).

The comparison of Cr(III) removal from various solutions is shown in Figure 8.

Not surprisingly, the maximum removal of Cr(III) was obtained from the single-component model solution and reached nearly 65%, while Cr(III) adsorption from the three-component solution was significantly lower (near 35%). As expected, the lowest percentage removal of Cr(III) was from the real solution. However, it must be emphasized that the 23% Cr(III) adsorbed means that 1 g of Cr(III) was retained in the column.

The reason for the worse percentage removal of Cr(III) from the three-component model solution than from the single-component solution is also the co-adsorption of Ni(II) and Co(II). The values for the percentage removal of these ions and the amount retained in the column are given in Table 8. Again, as was observed for batch adsorption, Dowex G26 appears to be a non-selective resin for the separation of divalent and trivalent metal ions from sulfate solutions. Moreover, divalent cobalt and nickel ions are adsorbed more efficiently than trivalent chromium, which leads to the loss of valuable Ni(II) and Co(II) from the sulfate solution. Furthermore, the desorption with 1.5 M H_2_SO_4_ appears to be a weak point in the systems studied (both model and real), as in one cycle (approximately 24 h), only 20–30% of the adsorbed Cr(III) can be removed from the resin and no more than 10% Ni(II) or 13% Co(II). This made it necessary to rinse the fixed bed with additional volumes of water and acid, which ultimately resulted in the generation of diluted solutions containing metal ions.

### 3.3. Cr(III) Removal by Precipitation 

In general, the dissolved Cr(III) ions can be removed by adjusting the pH to basic conditions, which converts Cr(III) into an insoluble hydroxide Cr(OH)_3_. As the adsorption results in both batch and column (continuous) experiments showed that selective removal of Cr(III) from the acidic solution is not possible without large losses of valuable Ni(II) and Co(II) in the system, the precipitation of Cr(OH)_3_ was investigated.

Precipitation was carried out with solutions of 3 or 30% NaOH, 10% CaO, 10% NaHCO_3_ and 10% Na_2_CO_3_ from real PLS (Table 1). The assumed pH values of the reactive mixture were as follows: 3, 4, 5. The changes in pH after reaching the required value just after precipitation (I) and 1 h after precipitation (F), as well as the amount of added solution, are shown in Table 9. Compared to NaOH and CaO, NaHCO_3_ and Na_2_CO_3_ solutions are required in much higher dosages to achieve the same pH because they are weaker alkalis. 

**Figure 9:**
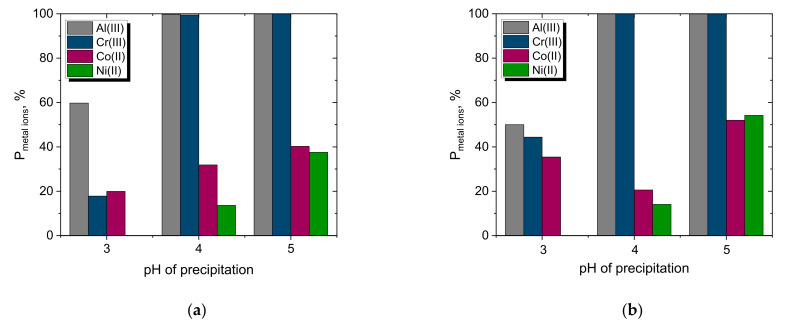

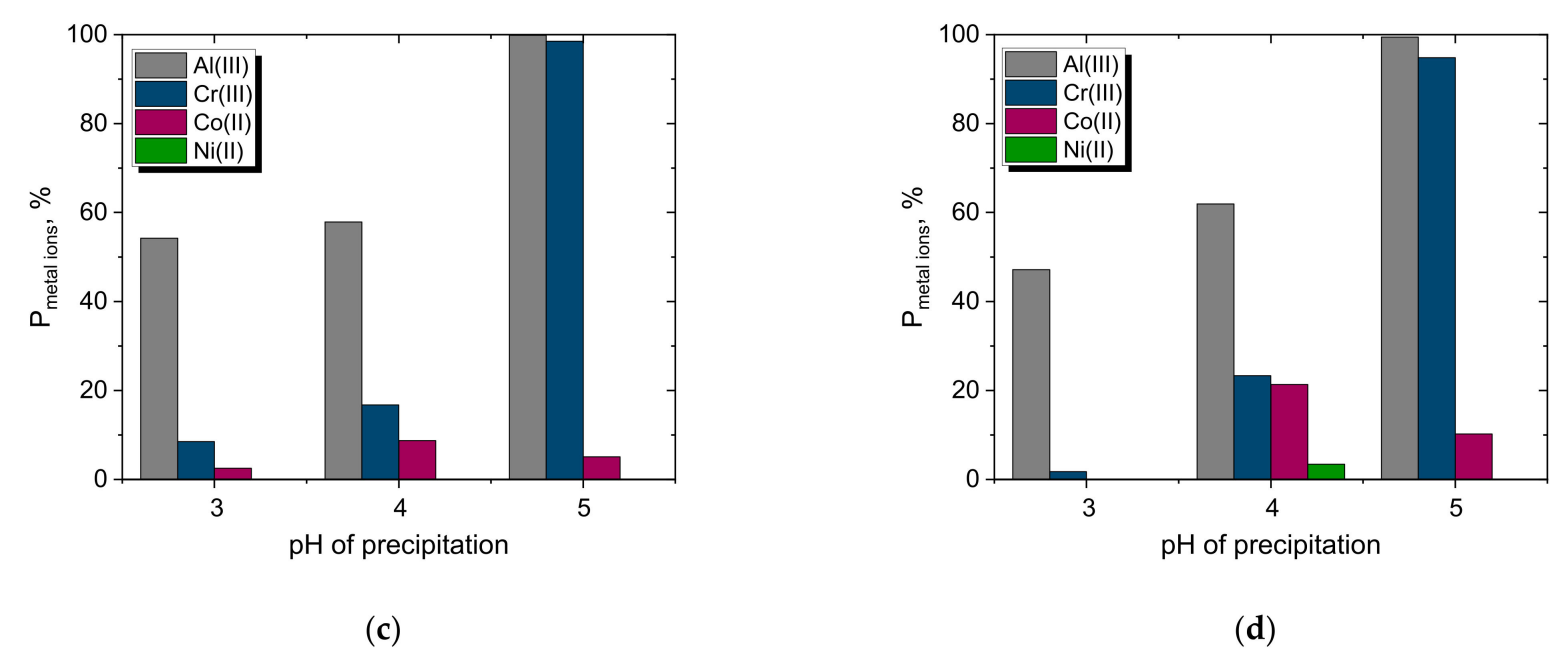
Yield of precipitation of Cr(III), Ni(II), Co(II) and Al(III) with solutions of (**a**) NaOH, (**b**) CaO, (**c**) NaHCO_3_, (**d**) Na_2_CO_3_ from the real PLS at the assumed pH values 3, 4 or 5.

The results of the precipitation yield of Cr(III), Ni(II) and Co(II) with various solutions are shown in Figure 9. Furthermore, Al(III) was also considered because it is present in real PLS, and the precipitation pH of Al(OH)_3_ is close to the pH value of Cr(III). For the known concentrations of Cr(III) and Al(III) in PLS, the pH of the precipitation of their hydroxides can be calculated based on the constants of the product solubility (K_sp,Cr(OH)3_ = 6.4·10^−31^_,_ K_sp,Al(OH)3_ = 1.3·10^−33^ at 25 °C [41]). Therefore, theoretically, Al(OH)_3_ should start to precipitate at pH 3.2, while Cr(OH)_3_ should precipitate at pH 4.1. The removal of Al(III) by co-precipitation with Cr(III) is profitable because of the removal of contamination that could negatively interfere during the next step of separation of Co(II) from Ni(II) by liquid–liquid extraction.

As expected, with increasing pH, the yield of precipitation increased. It was revealed that almost the entire amount of Cr(III) was precipitated and separated as a solid from the PLS. The precipitation yield exceeded 99% for the three reagents NaOH, CaO and NaHCO_3_ at pH 5. It should be noted that precipitation with the use of NaOH and CaO solutions led to a complete removal of Cr(III) and Al(III) at both pH 4 and 5, while metal ions, such as nickel and cobalt, remained mainly in the sulfate solution and precipitated to a much lower extent (P_Co(II)_ = 20–52%, P_Ni(II)_ = 0–54%) than the trivalent metals. 

Metal hydroxide precipitation with weak alkalis, i.e., NaHCO_3_ or Na_2_CO_3_, seemed to be even more advantageous than with NaOH solutions because the loss of precipitated Co(II) and Ni(II) was very small. The most selective solution for the precipitation of trivalent metal ions was the NaHCO_3_ solution. It precipitated no Ni(II) hydroxide and less than 10% Co(II), regardless of pH (Figure 9c). However, a much larger volume of the NaHCO_3_ solution was needed to obtain the assumed pH than NaOH. Therefore, despite such a good selectivity in precipitation with NaHCO_3_, it seems reasonable to precipitate Cr(III) and Al(III) with a NaOH solution but at pH 4, not 5, to reduce the loss of valuable Ni(II) and Co(II). NaOH is often applied for the precipitation of Cr(OH)_3_ from tannery wastewater [42,43,44], despite some disadvantages of its use, for example, poor sedimentation of the formed sludge [42]. Hence, it can be concluded that the NaOH solution is the best precipitating agent, out of the four reagents investigated, for Cr(III) removal from the sulfate solution, and from a technological point of view, the use of NaOH solutions as a pretreatment step is recommended.

## 4. Conclusions

The removal of chromium(III) ions from Ni-containing pregnant leach solutions can be an essential step to allow the effective deposition of nickel in the electrochemical system. The study showed that of the two proposed methods, adsorption (ion exchange) and precipitation, the second option is by far the most promising. The adsorption of Cr(III) was investigated for both model and real leach solutions, with two commercial cation exchange resins, i.e., Dowex G26 and MAC-3. Cr(III) precipitation was carried out from a real solution to compare its performance with that of adsorption and to choose the best operation for the preparation of an electrolyte for further steps of the hydrometallurgical process of nickel electrodeposition. 

Based on the results of the research, it can be concluded that precipitation is more efficient in the removal of Cr(III) from the solution and more selective toward the accompanying ions of valuable Ni(II) and Co(II). Of the resins used for single-component solutions, the Dowex G26 cationic sorbent, with a removal efficiency of up to 90% for chromium ions, is the more favorable option. However, in three-component or real solutions, this sorbent has a significantly higher sorption capacity for the divalent ions than for the trivalent ones. In the case of Cr(OH)_3_ precipitation, taking into account, on the one hand, the efficiency of the operation (the best results were obtained with the NaHCO_3_ solution) and, on the other hand, the amount of precipitating agent added and, consequently, the dilution of the solution (the smallest volume for a strong base, NaOH solution), the use of NaOH as a precipitating agent was the most favorable.

The research carried out indicates that precipitation is a suitable operation for pretreatment of Ni-containing waste solutions to prepare these solutions as a source for the modification of hydrogen storage alloys. This proposal is in line with current global trends regarding the use of post-processing waste as resources.

## Figures and Tables

**Table 1:** Composition of the PLS sulfate solution before and after DD: average concentration of metal ions and H^+^, Cl^−^, SO_4_^2−^.

Species	PLS before DD	PLS after DD
	Concentration in g/dm^3^	
Ni^2+^	26.7	37.2
Co^2+^	18.0	21.4
Cr^3+^	13.4	24.5
Al^3+^	7.20	7.60
Cu^2+^	0.020	0.020
Fe^3+^	0.138	0.132
Mg^2+^	0.023	0.021
Zn^2+^	0.022	0.021
Species	Concentration in M	
H^+^	4.10	3.46
SO_4_^2−^	2.28	n.a.
Cl^−^	0.19	0.16

n.a.—not applicable.

**Table 2:** Properties of the resins used for Cr(III) adsorption.

Property	Dowex G26	Dowex MAC-3
Matrix	Copolymer styrene-divinylbenzene	Polyacrylic, macroporous
Matrix active group	Sulfonic acid	Carboxylic acid
Form	Gel beads	White to amber opaque beads
Crosslinking	10%	n.a.
Moisture	45–52%	44–52%
Diameter	22–25 mesh 600–700 μm	16–50 mesh 300–1200 μm
Operating pH	0–14	n.a.
Character	Strongly acidic	Weakly acidic

n.a.—not applicable.

**Table 3:** Langmuir, Freundlich, Temkin and Dubinin–Radushkevich adsorption isotherm parameters.

Model	Parameters	Dowex G26	MAC-3
Langmuir	*Q_0_* (mg/g)	54.8	33.4
	*k_L_* (dm^3^/mg)	5.58 × 10^−5^	5.72 × 10^−8^
	*R* ^2^	0.995	0.993
Freundlich	*k_F_* (mg^1−1/n^·dm^3/n^/g)	0.062	4.73 × 10^−4^
	*n*	1.26	1.13
	*R* ^2^	0.973	0.997
Temkin	*A* (dm^3^/mg)	3.03 × 10^−3^	4.31 × 10^−4^
	*b_T_* (J g/mol mg)	380	104
	*R* ^2^	0.825	0.816
Dubinin–Radushkevich	*q_m_* (mg/g)	29.2	27.1
	*k_DR_* (mol^2^J^2^)	0.999	0.999
	*E* (kJ/mol)	0.707	0.707
	*R* ^2^	0.880	0.595

**Table 4:** The PFO and PSO kinetic parameters for Dowex G26 (abbreviation: initial concentration of Cr(III)_mass of sorbent_pH).

Parameter	2 g/dm^3^_	2 g/dm^3^_	2 g/dm^3^_	2 g/dm^3^_	2 g/dm^3^_	2 g/dm^3^_	10 g/dm^3^_	10 g/dm^3^_
	30 mg_	50 mg_	100 mg_	250 mg_	500 mg_	2000 mg_	30 mg_	50 mg_
	pH −0.5	pH −0.5	pH −0.5	pH −0.5	pH −0.5	pH −0.5	pH −0.5	pH −0.5
*k*_1_	1.05 × 10^−2^	9.37 × 10^−3^	9.74 × 10^−3^	5.13 × 10^−3^	5.04 × 10^−3^	5.30 × 10^−3^	1.22 × 10^−2^	9.69 × 10^−3^
*R* ^2^_k1_	0.788	0.731	0.773	0.781	0.956	0.890	0.781	0.774
*k*_2_	4.07 × 10^−4^	4.60 × 10^−4^	9.75 × 10^−4^	1.81 × 10^−3^	2.59 × 10^−3^	3.19 × 10^−2^	8.68 × 10^−5^	8.83 × 10^−5^
*R* ^2^_k2_	0.636	0.610	0.626	0.721	0.903	0.830	0.621	0.629
	**10 g/dm^3^_**	**10 g/dm^3^_**	**10 g/dm^3^_**	**10 g/dm^3^_**	**40 g/dm^3^_**	**40 g/dm^3^_**	**40 g/dm^3^_**	**40 g/dm^3^_**
	**100 mg_**	**250 mg_**	**500 mg_**	**2000 mg_**	**30 mg_**	**50 mg_**	**100 mg_**	**250 mg_**
	**pH −0.5**	**pH −0.5**	**pH −0.5**	**pH −0.5**	**pH −0.5**	**pH −0.5**	**pH −0.5**	**pH −0.5**
*k*_1_	8.31 × 10^−3^	5.78 × 10^−3^	1.21 × 10^−2^	6.15 × 10^−3^	1.01 × 10^−2^	8.39 × 10^−3^	1.32 × 10^−2^	6.16 × 10^−3^
*R* ^2^_k1_	0.727	0.848	0.677	0.921	0.781	0.718	0.769	0.913
*k*_2_	1.62 × 10^−4^	3.27 × 10^−4^	2.11 × 10^−3^	4.90 × 10^−3^	1.59 × 10^−5^	1.68 × 10^−5^	7.67 × 10^−5^	7.00 × 10^−5^
*R* ^2^_k2_	0.601	0.793	0.5926	0.858	0.637	0.599	0.603	0.876
	**40 g/dm^3^_**	**40 g/dm^3^_**	**2 g/dm^3^_**	**2 g/dm^3^_**	**2 g/dm^3^_**	**2 g/dm^3^_**	**2 g/dm^3^_**	**2 g/dm^3^_**
	**500 mg_**	**2000 mg_**	**250 mg_**	**500 mg_**	**2000 mg_**	**250 mg_**	**500_**	**2000_**
	**pH −0.5**	**pH −0.5**	**pH 2.2**	**pH 2.2**	**pH 2.2**	**pH 4.4**	**pH 4.4**	**pH 4.4**
*R* ^2^	0.879	0.973	0.970	0.966	0.955	0.969	0.984	0.962
*k*_1_	5.18 × 10^−3^	8.01 × 10^−3^	4.33 × 10^−3^	6.97 × 10^−3^	8.86 × 10^−3^	1.39 × 10^−2^	6.37 × 10^−3^	3.28 × 10^−3^
*R* ^2^_k1_	0.895	0.895	0.950	0.924	0.876	0.720	0.902	0.911
*k*_2_	1.25 × 10^−4^	1.14 × 10^−3^	1.88 × 10^−3^	6.02 × 10^−3^	0.132	2.18 × 10^−3^	7.13 × 10^−3^	3.83 × 10^−2^
*R* ^2^_k2_	0.835	0.818	0.901	0.875	0.809	0.621	0.837	0.846

where *k*_1_ in 1/min, *k*_2_ in g/mg∙min.

**Table 5:** The PFO and PSO kinetic parameters for MAC-3 (abbreviation: initial concentration of Cr(III)_mass of sorbent_pH).

Parameter	2 g/dm^3^_	2 g/dm^3^_	10 g/dm^3^_	10 g/dm^3^_	40 g/dm^3^_	40 g/dm^3^_	2 g/dm^3^_	2 g/dm^3^_	2 g/dm^3^_	2 g/dm^3^_
	250 mg_	500 mg_	250 mg_	500 mg_	250 mg_	500 mg_	250 mg_	500 mg_	250 mg_	500 mg_
	pH −0.5	pH −0.5	pH −0.5	pH −0.5	pH −0.5	pH −0.5	pH 2.2	pH 2.2	pH 4.4	pH 4.4
*R* ^2^	0.730	0.951	0.976	0.960	0.923	0.610	0.680	0.967	0.983	0.976
*k*_1_	5.35 × 10^−3^	7.19 × 10^−3^	5.55 × 10^−3^	6.87 × 10^−3^	7.48 × 10^−3^	9.52 × 10^−4^	1.10 × 10^−3^	3.98 × 10^−3^	3.65 × 10^−3^	3.10 × 10^−3^
*R* ^2^_k1_	0.664	0.889	0.904	0.959	0.961	0.222	0.358	0.897	0.935	0.924
*k*_2_	7.99 × 10^−2^	2.94 × 10^−2^	4.00 × 10^−2^	0.296	0.343	3.30 × 10^−3^	5.60 × 10^−2^	2.51 × 10^−2^	2.80 × 10^−3^	1.07 × 10^−3^
*R* ^2^_k2_	0.883	0.971	0.832	0.936	0.946	0.926	0.776	0.952	0.929	0.859

**Table 6:** Comparison of the performance of some cation exchange resins in Cr(III) adsorption.

Name of the Resin	Type of the Resin	Composition of the Feed	Cr(III) Removal, %	Adsorption Capacity, mg/g of the Resin	Ref.
Amberlite 200	Strongly acidic	0.3 g/dm^3^ Cr(III), pH 1	-	45.0	[24]
Amberlite IR120	Strongly acidic	0.008 g/dm^3^ Cr(III) in the presence of Al(III), Ni(II), pH 3.5 Cr(III) content not given, pH 2.8	18 -	- 10.1	[26] [38]
Lewatit TP207	Weakly acidic with chelating iminodiacetic acid groups	0.008 g/dm^3^ Cr(III) in the presence of Al(III), Ni(II), pH 3.5 0.05 g/dm^3^, pH 4.5	12 95	- 17.7	[26] [39]
Amberlite IRN77	Strongly acidic	0.005–0.05 g/dm^3^, pH 4 0.1 g/dm^3^, pH 3.5	95 100	18.2 23.9	[27] [31]
Amberlite IRN77-Fe_3_O_4_	Strongly acidic, coated with magnetite	0.1 g/dm^3^, pH 3.5	100	32.7	[31]
Purolite C160	Strongly acidic	0.005–0.05 g/dm^3^, pH 4	100	12.5	[27]
PVC-(SO_3_H)-(NH)	Polyampholyte, acidic and basic groups	0.5–13 g/dm^3^ Cr(III)	-	206	[37]
Dowex G26	Strongly acidic	2 g/dm^3^, pH −0.5	80.5	54.8	This work
MAC-3	Weakly acidic	2 g/dm^3^, pH −0.5	56.7	33.4	This work

**Table 7:** Removal of Cr(III), Ni(II) and Co(II) from three-component model solutions and the real PLS.

Time of Adsorption	Dowex G26								
	L/S = 1/250						L/S = 1/500		
	2 g/dm^3^ of Each Metal			10 g/dm^3^ of Each Metal			Real PLS		
	Cr(III)	Ni(II)	Co(II)	Cr(III)	Ni(II)	Co(II)	Cr(III)	Ni(II)	Co(II)
30	42.19	49.54	31.16	33.14	24.88	20.60	40.09	18.93	48.96
1080	-	-	-	-	-	-	63.76	55.59	71.96
1440	31.87	81.93	34.12	36.11	30.60	31.04	-	-	-
	**MAC-3**								
30	5.20	8.15	0.00	0.00	0.00	2.48	MAC-3 not used		
1440	7.74	16.08	3.20	9.04	11.58	15.33			

**Table 8:** Adsorption of Cr(III), Ni(II) and Co(II) from the model and real solutions after 24 h and percentage desorption in one cycle with 100 cm^3^ 1.5 M H_2_SO_4_.

Type of Solution	Removal, %			Amount Adsorbed in the Column, g			Desorption, %		
	Cr(III)	Ni(II)	Co(II)	Cr(III)	Ni(II)	Co(II)	Cr(III)	Ni(II)	Co(II)
Model 1	64.73	-	-	0.125	-	-	21.43	-	-
Model 3	35.77	49.20	50.64	0.093	0.086	0.100	32.00	9.20	8.02
Real PLS after DD	22.61	48.70	39.08	1.04	3.55	1.63	20.00	7.40	12.30

Model 1—single-component model solution (1 g/dm^3^ Cr(III), 3 M H^+^); Model 3—three-component model solution (1 g/dm^3^ Cr(III), 1 g/dm^3^ Ni(II), 1 g/dm^3^ Co(II), 3 M H^+^); Real PLS after DD—(24.5 g/dm^3^ Cr(III), 37.2 g/dm^3^ Ni(II), 21.4 g/dm^3^ Co(II), 3.46 M H^+^).

**Table 9:** pH after reaching the required value and after an hour, and the amount of solution added.

Assumed pH	Meas.	NaOH (3 or 30%)		NaHCO_3_ (10%)		Na_2_CO_3_ (10%)		CaO (10%)	
		pH_m_	V, cm^3^	pH_m_	V, cm^3^	pH_m_	V, cm^3^	pH_m_	V, cm^3^
pH 3	I	3.58	16.5 (30%)	2.95	95	2.97	70.5	2.87	50
	F	3.56		3.01		3.07		3.84	
pH 4	I	4.04	18 (30%) + 22 (3%)	3.97	118.5	3.99	88.5	3.97	64
	F	4.12		3.87		3.91		6.36	
pH 5	I	5.60	23 (30%) + 2 (3%)	5.03	136	4.96	99.5	5.01	72.5
	F	5.59		4.87		4.84		7.77	

I—initial pH, just after precipitation; F—final pH, after 1 h; pH_m_—measured pH; V—volume of the precipitating solution.

## Data Availability

The data presented in this study are available on request from the corresponding author.

## Appendix A

**Table A1:** *a*–*d* parameters of the fitted equation for Dowex G26 (abbreviation: initial concentration of Cr(III)_mass of sorbent_pH).

Coefficient	2 g/dm^3^_	2 g/dm^3^_	2 g/dm^3^_	2 g/dm^3^_	2 g/dm^3^_	2 g/dm^3^_	10 g/dm^3^_	10 g/dm^3^_
	30 mg_	50 mg_	100 mg_	250 mg_	500 mg_	2000 mg_	30 mg_	50 mg_
	pH −0.5	pH −0.5	pH −0.5	pH −0.5	pH −0.5	pH −0.5	pH −0.5	pH −0.5
*a*	−2.00 × 10^−4^	12.7	−5.10 × 10^−4^	−9.09 × 10^−6^	−9.72 × 10^−6^	−8.31 × 10^−6^	−9.51	0.156
*b*	−1.41	0.382	−0.283	−3.66	−3.21	−3.56	−1.81	0.657
*c*	10.5	0.808	10.6	10.9	10.6	8.16	1.06	6.36
*d*	45.4	−11.3	34.0	13.0	7.55	1.80	225	−9.83
*R* ^2^	0.953	0.861	0.991	0.865	0.996	0.955	0.975	0.951
	**10 g/dm^3^_**	**10 g/dm^3^_**	**10 g/dm^3^_**	**10 g/dm^3^_**	**40 g/dm^3^_**	**40 g/dm^3^_**	**40 g/dm^3^_**	**40 g/dm^3^_**
	**100 mg_**	**250 mg_**	**500 mg_**	**2000 mg_**	**30 mg_**	**50 mg_**	**100 mg_**	**250 mg_**
	**pH −0.5**	**pH −0.5**	**pH −0.5**	**pH −0.5**	**pH −0.5**	**pH −0.5**	**pH −0.5**	**pH −0.5**
*a*	24.0	−5.76 × 10^−5^	−5.69 × 10^−10^	−7.68 × 10^−6^	2.16	1.16	−14.3	−999
*b*	0.593	−3.65	−24.2	−4.09	0.760	0.936	−1.73	−6.88
*c*	0.678	10.8	9.33	9.95	5.28	5.37	1.03	−6.45
*d*	3.66	37.2	17.8	6.78	−11.0	−10.6	267	105
*R* ^2^	0.977	0.892	0.807	0.985	0.896	0.924	0.992	0.814
	**40 g/dm^3^_**	**40 g/dm^3^_**	**2 g/dm^3^_**	**2 g/dm^3^_**	**2 g/dm^3^_**	**2 g/dm^3^_**	**2 g/dm^3^_**	**2 g/dm^3^_**
	**500 mg_**	**2000 mg_**	**250 mg_**	**500 mg_**	**2000 mg_**	**250 mg_**	**500_**	**2000_**
	**pH −0.5**	**pH −0.5**	**pH 2.2**	**pH 2.2**	**pH 2.2**	**pH 4.4**	**pH 4.4**	**pH 4.4**
*a*	−118	−1.19 × 10^−5^	−1.20 × 10^−4^	−3.09 × 10^−6^	−6.05	−228	−3.21 × 10^−6^	−7.11 × 10^−6^
*b*	−5.38	−5.54	−2.54	−4.51	−7.24	−33.7	−4.50	−2.35
*c*	−4.02	10.4	8.70	10.5	−8.40	−22.7	10.3	8.39
*d*	60.9	27.0	11.8	6.13	2.08	13.9	7.63	1.94
*R* ^2^	0.879	0.973	0.970	0.966	0.955	0.969	0.984	0.962

**Table A2:** *a*–*d* parameters of the fitted equation for MAC-3 (abbreviation: initial concentration of Cr(III)_mass of sorbent_pH).

Coefficient	2 g/dm^3^_	2 g/dm^3^_	10 g/dm^3^_	10 g/dm^3^_	40 g/dm^3^_	40 g/dm^3^_	2 g/dm^3^_	2 g/dm^3^_	2 g/dm^3^_	2 g/dm^3^_
	250 mg_	500 mg_	250 mg_	500 mg_	250 mg_	500 mg_	250 mg_	500 mg_	250 mg_	500 mg_
	pH −0.5	pH −0.5	pH −0.5	pH −0.5	pH −0.5	pH −0.5	pH 2.2	pH 2.2	pH 4.4	pH 4.4
*a*	−3.86 × 10^−5^	−1.35 × 10^−5^	−1.50 × 10^−4^	−2.00 × 10^−4^	−1.60 × 10^−4^	12.3	−1.50 × 10^−4^	−7.73 × 10^−5^	−1.90 × 10^−4^	−2.30 × 10^−4^
*b*	−3.67	−4.74	−3.80	−3.11	−4.77	0.278	−0.818	−2.93	−2.55	−2.26
*c*	9.71	9.64	10.0	9.30	10.6	1.47	10.2	9.23	9.01	8.48
*d*	10.0	5.45	30.9	15.20	118	−10.3	8.16	4.04	9.91	5.84
*R* ^2^	0.730	0.951	0.976	0.960	0.923	0.610	0.680	0.967	0.983	0.976
